# Relationship between Body Mass Index, C-Peptide, and Delta-5-Desaturase Enzyme Activity Estimates in Adult Males

**DOI:** 10.1371/journal.pone.0149305

**Published:** 2016-03-29

**Authors:** C. Austin Pickens, Karen H. Matsuo, Jenifer I. Fenton

**Affiliations:** Department of Food Science and Human Nutrition, Michigan State University, East Lansing, MI, 48824, United States of America; East Tennessee State University, UNITED STATES

## Abstract

Obesity, in particular abdominal obesity, alters the composition of plasma and tissue fatty acids (FAs), which contributes to inflammation and insulin resistance. FA metabolism is modulated by desaturases and may affect adipokine and insulin secretion. Therefore, we examined relationships between adipokines, a marker of insulin production, and plasma FA desaturase enzyme activity estimates (EAEs) in obesity. Plasma phospholipid (PPL) FAs were isolated from 126 males (ages 48 to 65 years), derivatized, and analyzed using gas chromatography. Delta-6 desaturase (D6D) and delta-5 desaturase (D5D) EAEs were calculated as the ratio of PPL 20:3/18:2 and 20:4/20:3, respectively. In body mass index (BMI) and waist circumference (WC) adjusted polytomous logistic regression analyses, PPL FAs and FA desaturase EAEs were associated with C-peptide and adiponectin. Individuals with elevated D6D EAEs were less likely (OR 0.33) to have serum adiponectin concentrations > 5.37 μg/mL, compared with adiponectin concentrations ≤ 3.62 μg/mL. Individuals with increased D5D EAEs were less likely (OR 0.8) to have C-peptide concentrations ≥ 3.32 ng/mL, and > 1.80 and ≤ 3.29 ng/mL, compared with those with C-peptide ≤ 1.76 ng/mL. The proinflammatory cytokine tumor necrosis factor-α (TNF- α) was positively associated with C-peptide, but TNF- α was not associated with the D5D EAE. C-peptide and adiponectin concentrations are associated with specific PPL FAs and FA desaturase EAEs. The relationship between C-peptide concentrations and D5D EAEs remained significant after adjusting for BMI, WC, and TNF-α. Thus, future research should investigate whether D5D inhibition may occur through a C-peptide mediated pathway.

## Introduction

Obesity is a chronic disease affecting over one-third of US adults [1]. Obesity is associated with excess lipid storage in white adipose tissue (WAT), adipokine dysregulation, insulin resistance, and chronic low-grade inflammation [2]. Adipokines are adipose-derived cytokines, which have functions in regulating metabolism and inflammation. The expansion of WAT alters adipokine secretion and fatty acid (FA) metabolism, and also influences low-grade inflammation associated with insulin resistance and type-2 diabetes (T2D) [3]. In obesity, circulating concentrations of anti-inflammatory adipokines are lower (i.e. adiponectin) and pro-inflammatory adipokines (i.e. leptin) are elevated compared with lean individuals [4].

Adipokines are necessary for normal cellular function, but dysregulated adipokine secretion can have pathological effects. Leptin is important for regulating body fat [2], but in obesity, leptin concentrations are elevated and individuals can become “leptin resistant” resulting in increased weight gain [reviewed in detail [5]]. Leptin and adiponectin concentrations have an inverse relationship in obese individuals. Adiponectin is an adipokine that increases FA oxidation and glucose utilization in tissues [6]. In obesity-associated insulin resistance, adiponectin concentrations are lower and adiponectin receptors are downregulated [7]. C-peptide, a protein cleaved from pro-insulin, is inversely associated with adiponectin, and C-peptide is positively associated with leptin secretion [8]. While C-peptide is not an adipokine, it is used as a biomarker of insulin secretion which is altered in obesity [9]. Increases in several plasma FAs trigger inflammation, which contributes to insulin resistance and results in increased C-peptide concentrations. There is a relationship between FAs, obesity, adipokines, and insulin resistance, however, it is unknown whether adipokines are associated with specific FAs.

FAs are classified into 3 categories: saturated FAs (SatFAs), monounsaturated FAs (MUFAs), and polyunsaturated FAs (PUFAs). PUFAs can be of the omega-3 (ω-3) or omega-6 (ω-6) family, and obese individuals tend to have lower blood concentrations of ω-3s and greater blood concentrations of ω-6s [10]. FAs such as PUFAs are obtained through dietary intake, or endogenously synthesized by elongating and desaturating enzymes. Obesity-associated inflammation may alter enzyme activity and this altered enzymatic expression can modify lipid metabolism [11]. For instance, obese individuals with insulin resistance have decreased expression of the enzyme delta-5-desaturase (D5D) in skeletal muscle [12]. Obesity is also associated with lipid changes such as increased plasma SatFAs [13], in particular, palmitic acid (PA) and stearic acid (SA) [14]. Elevated circulating concentrations of SatFAs can increase inflammation and affect secretion of pro-inflammatory cytokines [15], in particular, tumor necrosis factor-α (TNF-α) which impairs insulin receptor downstream signaling [16]. Because FAs may influence adipokine secretion and insulin resistance, determining associations between FAs and FA desaturase enzymes, adipokines, and markers of insulin production may lead to a better understanding of obesity-associated pathologies and lead to discovery of potential therapeutic targets.

Most studies investigating the role of lipids in obesity focus on altered dietary FA intake [13] or red blood cell FA concentrations [17]. Similarly, numerous studies investigate the association of obesity with adipokines or insulin resistance [3, 18]. However, there is limited information on specific plasma FA associations with adiponectin, leptin, and C-peptide after adjusting (i.e. statistically) for obesity. Determining whether changes in FAs and FA metabolism occur independent of obesity and abdominal adiposity may allude to unknown biological relationships between FAs and adipokine dysregulation. Therefore, in this study we examined the relationship between C-peptide, adiponectin, leptin, and plasma FAs and FA desaturase enzyme activity estimates (EAEs) in overweight adults.

## Materials and Methods

### Ethics Statement

The study was approved by the Biomedical and Health Institutional Review Board of Michigan State University (IRB# 08–786). The Biomedical and Health Institutional Review Board is one of three IRB committees on the Michigan State University East Lansing campus. Michigan State University's IRBs were established to advance the goal of conducting research with diligence and integrity. The purpose of the committee is to protect the rights, welfare and privacy of human subjects who participate in research conducted by students and/or faculty affiliated with MSU. At the time of enrollment, written informed consent was obtained from each participant.

### Study Population

Male subjects (n = 126, >96% Caucasian) 50–65 years of age were previously recruited from the Tri-County Gastroenterology P.C., Clinton, MI, as previously reported [19]. In brief, nurses at the GI center were responsible for recruitment, explaining the study, and obtaining consent. Patient exclusion was performed, as previously reported [19]. In brief, patients were excluded for: 1) current immunosuppressants or antibiotics, 2) allergic disorders such as eosinophilic or mast cell disorders, severe asthma, 3) severe co-morbidities like end stage renal disease or liver disease with cirrhosis, autoimmune illness, chronic hepatitis, other chronic infections, 4) diabetes. At time of enrollment, trained staff collected anthropometric measurements and venous blood of study participants. These measurements were used to calculate BMI (kg/m^2^). Patients reported fasting before venous blood was collected, so lipid results will not be altered by dietary intake. Study participants were classified as lean (BMI <25), overweight (25≤ BMI <30), or obese (BMI ≥30). Serum and plasma fractions were separated from venous blood after collection, stored at -80°C. In addition, the samples were blinded and coded so there are no unique identifiers. Clinical metadata on subject co-morbidities, current medications, family history and tumor characteristics was also collected.

### Serum Adipokine and C-peptide Analysis

Adipokines and C-peptide were analyzed using ELISA or multiplex cytokine kits as previously reported [20]. A commercially available leptin ELISA kit was performed per manufacturer’s instructions (R&D Systems, DY398; Minneapolis, MN). C-peptide concentrations were measured as directed by the manufacturer (Calbiotech, Spring Valley, CA, REF; CP1795). Total adiponectin measurements were performed following the manufacturer’s instructions (Alpco Diagnostics, Salem, NH).

### Plasma Phospholipid Extraction, Isolation and Analysis

In brief, approximately 200 mg plasma per subject was weighed and extracted using a modified Rose and Oaklander extraction [21]. Phospholipids (PLs) were isolated using Isolute-XL ® SPE aminopropyl columns (500 mg; Bioatage, Charlotte, NC) as described by Agren et al [22]. Fatty acid methyl esters (FAMEs) were prepared as previously described [23, 24]. Plasma PL (PPL) FAMEs were analyzed using HS-Omega-3 Index® methodology at OmegaQuant Analytics, LLC (Sioux Falls, SD) as previously described [25].

### Statistical analyses

Frequencies, means, and standard deviations were calculated for descriptive analyses (Table 1). Each FA was expressed as a percentage of total PPL. FA enzyme activity estimates (EAE) were calculated as the ratio of product-to-substrate for delta-5-desaturase (D5D) and delta-6-desaturase (D6D) as follows: D5D = arachidonic (AA)/dihomo-γ-linolenic acid (DGLA); D6D = DGLA/linoleic acid (LA). The total PPL ω-3, herein referred to as total ω-3, was calculated as ∑ alpha-linolenic acid (ALA) + eicosapentaenoic acid (EPA) + docosapentaenoic acid ω-3 (DPAω-3) + docosahexaenoic acid (DHA); The total PPL ω-6, herein referred to as total ω-6, was calculated as ∑ LA + linoelaidic + DGLA + AA + docosatetraenoic acid (DTA) + DPAω-6; ω-6/ω-3 ratio was calculated as total ω-6/ total ω-3. Both Pearson and Spearman correlations were performed to correlate C-peptide, adipokines, and FAs and EAEs, but since several variables were non-normally distributed, Spearman correlations are presented (S1 Table).

**Table 1:** Age, anthropometric, serum adipokines and insulin marker, and plasma phospholipid fatty acid percentages^a^.

	Overall
*n*	126
Age (years)	56.9 ± 4.7
Age range ^b^	(48–65)
BMI (kg/m^2)	29.7 ± 5.2
BMI range ^b^	(19.20–45.57)
WC (inches)	41.4 ± 6.1
WC range ^b^	(29.75–57.50)
Leptin (ng/mL)	9.9 ± 10.5
Leptin range ^b^	(0.31–49.47)
Adiponectin (μg/mL)	4.8 ± 2.3
Adiponectin range ^b^	(1.02–13.20)
C-peptide (ng/mL)	2.9 ± 1.8
C-peptide range ^b^	(0.72–9.90)
PA	29.2 ± 3.1
SA	14.5 ± 2.2
LGCA	1.6 ± 0.5
NA	0.4 ± 0.1
ALA	0.2 ± 0.4
EPA	0.8 ± 0.7
DHA	3.0 ± 1.3
DPAω-3	
total ω-3	4.9 ± 2.0
LA	19.6 ± 3.7
DGLA	2.7 ± 0.9
AA	10.3 ± 2.8
DTA	0.4 ± 0.1
DPAω-6	0.3 ± 0.1
total ω-6	34.1 ± 3.9
ω-6:ω-3	8.1 ± 3.0
D5D	4.1 ± 1.6
D6D	0.14 ± 0.05

^a^ Values expressed as mean ± standard deviation unless otherwise noted. PPL measurements expressed as percentage of total.

^b^ Values listed in parenthesis expressed as range of values corresponding to age, BMI, WC, leptin, adiponectin, and C-peptide concentrations, respectively.

Odds ratios (OR) and 95% confidence intervals were calculated using polytomous logistic regression models for categorical outcome data. Categories were defined by dividing the population into tertiles based on serum adipokine and C-peptide concentration. In all logistic regression models, adipokines and C-peptide were analyzed categorically as dependent variables, with the reference category defined as individuals in the first tertile; or the third of population with the lowest concentration leptin, adiponectin, and C-peptide, respectively. FAs were analyzed as continuous independent variables in logistic regression models. We have previously reported age is associated with several FAs and EAEs [24], therefore, all models were adjusted for age. Due to high correlation (>0.9, data not shown) between body mass index (BMI) and waist circumference (WC), these anthropometric measurements could not be analyzed in the same model. Instead, two additional models were run, the first with the addition of BMI and the second with the addition of WC. The odds ratios for ALA, DTA, DPA ω-6, and D6D EAEs were calculated on the basis that there is a unit change of 0.1 for the respective beta coefficient for each given parameter. Statistically significant when p ≤ 0.05 and a statistical trend was defined as 0.05 < p ≤ 0.09. Statistical analyses were conducted using SAS version 9.3 (Cary, NC).

## Results

Participant characteristics and FA concentrations are displayed in Table 1. Serum adiponectin concentrations were significantly associated with PPL FAs (Tables 2 and 3). Lignoceric acid (LGCA) was the only FA significantly associated with serum adiponectin concentrations in the second tertile, > 3.64 and ≤ 5.37 μg/mL, when compared with individuals with adiponectin concentrations in the first tertile, ≤ 3.62 μg/mL (Table 2). For each unit increase in LGCA, individuals were approximately 3 times more likely to have serum adiponectin concentrations in the second tertile compared with the first tertile in all models. LGCA was not associated with serum adiponectin in the third tertile. Serum adiponectin concentrations in the third tertile, > 5.37 μg/mL, were significantly associated with several FAs across logistic regression models when compared with individuals with adiponectin concentrations in the first tertile, ≤ 3.62 μg/mL (Table 3). The only SatFA associated with the third tertile of adiponectin was PA. For each unit increase in PA, individuals were approximately 0.80 times as likely to have serum adiponectin concentrations in the third tertile compared with the first tertile in all models (Table 3).

**Table 2:** The second tertile of serum adiponectin concentrations is significantly associated with PPL LGCA^a^.

	Model 1 Age		Model 2 Age + BMI		Model 3 Age + WC	
FA/EAE	OR (95% CI)	p-value	OR (95% CI)	p-value	OR (95% CI)	p-value
PA	0.99 (0.86, 1.12)	0.830	1.00 (0.87, 1.15)	0.981	1.00 (0.87, 1.14)	0.962
SA	0.95 (0.79, 1.15)	0.629	0.98 (0.80, 1.19)	0.800	0.98 (0.74, 1.19)	0.831
LGCA	3.21 (1.20, 8.56)	**0.020**	2.86 (1.06, 7.74)	**0.038**	2.92 (1.09, 7.82)	**0.033**
NA	0.90 (0.39, 2.04)	0.791	0.75 (0.32, 1.76)	0.507	0.74 (0.31, 1.76)	0.497
ALA ^b^	1.01 (0.67, 1.54)	0.958	0.97 (0.64, 1.48)	0.890	0.98 (0.64, 1.49)	0.918
EPA	2.02 (0.91, 4.47)	*0*.*085*	1.84 (0.84, 4.00)	0.126	1.81 (0.82, 4.01)	0.142
DHA	1.23 (0.88, 1.72)	0.227	1.19 (0.85, 1.68)	0.312	1.18 (0.83, 1.67)	0.348
Total ω-3	1.19 (0.95, 1.50)	0.130	1.16 (0.92, 1.47)	0.213	1.16 (0.91, 1.47)	0.231
LA	1.06 (0.93, 1.20)	0.382	1.04 (0.91, 1.18)	0.579	1.04 (0.91, 1.18)	0.550
DGLA	0.85 (0.52, 1.41)	0.529	0.99 (0.58, 1.69)	0.963	0.96 (0.56, 1.65)	0.885
AA	0.97 (0.84, 1.13)	0.703	0.97 (0.83, 1.14)	0.716	0.98 (0.84, 1.14)	0.763
DTA ^b^	0.30 (0.01, 6.55)	0.447	0.59 (0.02, 15.06)	0.750	0.47 (0.02, 12.23)	0.647
DPA ω-6 ^b^	0.88 (0.63, 1.21)	0.418	0.95 (0.68, 1.34)	0.768	0.93 (0.66, 1.30)	0.658
Total ω-6	1.01 (0.91, 1.13)	0.806	1.01 (0.90, 1.12)	0.933	1.01 (0.91, 1.13)	0.864
ω-6:ω-3	0.98 (0.84, 1.13)	0.739	0.99 (0.85, 1.15)	0.871	1.00 (0.85, 1.16)	0.952
D5D	0.97 (0.73, 1.28)	0.819	0.91 (0.69, 1.21)	0.518	0.93 (0.70, 1.23)	0.591
D6D ^b^	0.66 (0.30, 1.46)	0.305	0.82 (0.35, 1.93)	0.653	0.78 (0.33, 1.85)	0.577

^a^ Models defined as: Adiponectin = fatty acid + independent variable(s) next to model number. Fatty acids expressed as percent of total phospholipids. P-values bolded if p ≤ 0.05 and italicized if 0.05 < p ≤0.09.

^b^ Odds ratios calculated on the basis that there is a unit change of 0.1 for the respective beta coefficient for each given parameter.

**Table 3:** The third tertile of serum adiponectin concentrations is significantly associated with PPL PA, LA, Total ω-6, and D6D EAE^a^.

	Model 1 Age		Model 2 Age + BMI		Model 3 Age + WC	
Fatty Acid	OR (95% CI)	p-value	OR (95% CI)	p-value	OR (95% CI)	p-value
PA	0.78 (0.65, 0.93)	**0.006**	0.82 (0.68, 0.99)	**0.037**	0.82 (0.68, 0.99)	**0.035**
SA	0.85 (0.69, 1.05)	0.129	0.90 (0.72, 1.11)	0.321	0.92 (0.74, 1.15)	0.461
LGCA	1.55 (0.57, 4.22)	0.388	1.24 (0.44, 3.53)	0.687	1.26 (0.44, 3.55)	0.668
NA	1.66 (0.75, 3.69)	0.213	1.18 (0.49, 2.84)	0.714	1.05 (0.43, 2.58)	0.911
ALA ^b^	1.33 (0.92, 1.92)	0.13	1.25 (0.85, 1.84)	0.261	1.24 (0.84, 1.81)	0.281
EPA	1.60 (0.70, 3.64)	0.265	1.32 (0.57, 3.09)	0.519	1.13 (0.48, 2.69)	0.777
DHA	0.99 (0.69, 1.41)	0.949	0.91 (0.63, 1.33)	0.639	0.87 (0.59, 1.27)	0.463
Total ω-3	1.12 (0.89, 1.41)	0.349	1.03 (0.80, 1.33)	0.797	1.00 (0.77, 1.28)	0.979
LA	1.27 (1.10, 1.45)	**0.001**	1.20 (1.04, 1.39)	**0.011**	1.20 (1.04, 1.39)	**0.011**
DGLA	0.62 (0.36, 1.04)	*0*.*069*	0.84 (0.48, 1.48)	0.547	0.86 (0.48, 1.52)	0.598
AA	0.93 (0.80, 1.09)	0.361	0.95 (0.81, 1.12)	0.515	0.96 (0.82, 1.13)	0.649
DTA ^b^	0.04 (0.001, 0.92)	**0.044**	0.16 (0.01, 5.20)	0.304	0.16 (0.01, 4.98)	0.294
DPA ω-6 ^b^	0.67 (0.46, 0.97)	**0.033**	0.80 (0.54, 1.18)	0.255	0.79 (0.54, 1.17)	0.243
Total ω-6	1.15 (1.01, 1.30)	**0.031**	1.13 (0.99, 1.29)	*0*.*070*	1.14 (1.01, 1.30)	**0.049**
ω-6:ω-3	1.05 (0.91, 1.21)	0.546	1.08 (0.92, 1.26)	0.34	1.11 (0.95, 1.29)	0.207
D5D	1.12 (0.86, 1.46)	0.395	1.02 (0.77, 1.34)	0.991	1.03 (0.78, 1.36)	0.827
D6D ^b^	0.20 (0.08, 0.53)	**0.001**	0.33 (0.12, 0.90)	**0.031**	0.33 (0.12, 0.92)	**0.034**

^a^ Models defined as: Adiponectin = fatty acid + independent variable(s) next to model number. Fatty acids expressed as percent of total phospholipids. P-values bolded if p ≤ 0.05 and italicized if 0.05 < p ≤0.09.

^b^ Odds ratios calculated on the basis that there is a unit change of 0.1 for the respective beta coefficient for each given parameter.

PPL ω-6 PUFAs were not associated with adiponectin concentrations in the second tertile (Table 2). However, PPL ω-6 PUFAs were significantly associated with adiponectin concentrations in the third tertile (Table 3). Most ω-6 PUFAs were inversely associated with the third adiponectin tertile when analyzed individually, except LA (Table 3). For each unit increase in LA, individuals were approximately 1.20 times more likely to have serum adiponectin concentrations in the third tertile compared with the first tertile across all models. Total ω-6 was positively associated with adiponectin (S1 Table). For each unit increase in total ω-6, individuals were approximately 1.14 times more likely to have serum adiponectin concentrations in the third tertile rather than the first, in all models (Table 3). The D6D EAE was inversely associated with adiponectin (Table 3). Specifically, for each unit increase in D6D, individuals were 0.20 (0.08, 0.53) times as likely in the age-adjusted model, and 0.33 times as likely in the BMI- and WC-adjusted models, to have serum adiponectin concentrations in the third tertile compared with the first.

Serum C-peptide concentrations in the second tertile, > 1.80 and ≤ 3.29 ng/mL, were also significantly associated with FAs, compared with C-peptide concentrations in the first tertile, ≤ 1.76 ng/mL (Table 4). Generally, PPL PUFAs were significantly associated with C-peptide only after adjusting for BMI or WC (Table 4). For each unit increase in the ω-3 PPL ALA, individuals were 1.56 (1.01. 2.43) times more likely in the WC-adjusted model to have serum C-peptide concentrations in the second tertile compared with the first tertile. The ω-6s were also significantly associated with C-peptide only after adjusting for BMI and WC. For each unit increase in PPL AA, individuals were approximately 0.80 times as likely in the BMI- and WC-adjusted models, to have serum C-peptide concentrations in the second tertile compared with the first tertile. For each unit increase in PPL DTA, individuals were 0.70 (0.49, 0.99) times as likely in the BMI-adjusted model, and 0.62 (0.42, 0.93) times as likely in the WC-adjusted model, to have serum C-peptide concentrations in the second tertile rather than the first. The D5D EAE was inversely associated with C-peptide across all logistic regression models. For each unit increase in D5D, individuals were approximately 0.69 times as likely to have serum C-peptide concentrations in the second tertile compared with the first tertile, across all models.

**Table 4:** The second tertile of serum C-peptide concentrations is significantly associated with PPL AA, DTA, and D5D EAE^a^.

	Model 1 Age		Model 2 Age + BMI		Model 3 Age + WC	
Fatty Acid	OR (95% CI)	p-value	OR (95% CI)	p-value	OR (95% CI)	p-value
PA	1.11 (0.95, 1.29)	0.188	1.02 (0.87, 1.19)	0.791	1.01 (0.87, 1.18)	0.871
SA	1.25 (1.01, 1.55)	**0.043**	1.19 (0.96, 1.48)	0.116	1.14 (0.90, 1.42)	0.276
LGCA	0.51 (0.21, 1.26)	0.146	0.56 (0.21, 1.44)	0.227	0.54 (0.20, 1.44)	0.218
NA	0.36 (0.15, 0.83)	**0.017**	0.45 (0.17, 1.15)	0.095	0.56 (0.21, 1.49)	0.244
ALA ^b^	1.19 (0.85, 1.67)	0.302	1.40 (0.94, 2.07)	0.099	1.56 (1.01, 2.43)	**0.048**
EPA	0.75 (0.38, 1.51)	0.866	0.92 (0.44, 1.92)	0.832	1.20 (0.55, 2.60)	0.645
DHA	0.90 (0.64, 1.27)	0.537	0.98 (0.69, 1.41)	0.922	1.04 (0.72, 1.51)	0.817
Total ω-3	0.97 (0.78, 1.20)	0.767	1.07 (0.85, 1.35)	0.549	1.14 (0.90, 1.45)	0.285
LA	0.96 (0.85, 1.08)	0.489	1.04 (0.91, 1.18)	0.576	1.05 (0.93, 1.20)	0.433
DGLA	1.62 (0.95, 2.76)	*0*.*078*	1.19 (0.67, 2.11)	0.550	1.07 (0.59, 1.95)	0.823
AA	0.87 (0.74, 1.02)	*0*.*085*	0.81 (0.67, 0.97)	**0.019**	0.77 (0.63, 0.93)	**0.006**
DTA ^b^	0.90 (0.66, 1.23)	0.503	0.70 (0.49, 0.99)	**0.047**	0.62 (0.42, 0.93)	**0.020**
DPA ω-6 ^b^	1.28 (0.88, 1.86)	0.189	1.08 (0.72, 1.61)	0.721	1.02 (0.68, 1.54)	0.919
Total ω-6	0.90 (0.80, 1.02)	0.102	0.91 (0.80, 1.04)	0.162	0.90 (0.78, 1.03)	0.113
ω-6:ω-3	1.02 (0.88, 1.17)	0.832	0.98 (0.85, 1.14)	0.818	0.95 (0.82, 1.11)	0.547
D5D	0.67 (0.50, 0.91)	**0.009**	0.70 (0.51, 0.94)	**0.019**	0.69 (0.51, 0.94)	**0.019**
D6D ^b^	2.47 (1.02, 5.99)	**0.045**	1.32 (0.52, 3.39)	0.559	1.09 (0.41, 2.86)	0.869

^a^ Models defined as: C-peptide = fatty acid + independent variable(s) next to model number. Fatty acids expressed as percent of total phospholipids. P-values bolded if p ≤ 0.05 and italicized if 0.05 < p ≤0.09.

^b^ Odds ratios calculated on the basis that there is a unit change of 0.1 for the respective beta coefficient for each given parameter.

Serum C-peptide concentrations in the third tertile, ≥ 3.32 ng/mL, were also significantly associated with FAs, compared with C-peptide concentrations in the first tertile, ≤ 1.76 ng/mL, (Table 5). No PPL SatFAs were associated with the third tertile of C-peptide. PPL ω-3 PUFAs were not associated with the third tertile of C-peptide. AA was the only ω-6 PUFA associated with C-peptide after adjusting for BMI and WC. For each unit increase in AA, individuals were approximately 0.80 times as likely in the BMI- and WC-adjusted models, to have serum C-peptide concentrations in the third tertile compared with the first tertile, respectively. The D5D EAE was inversely associated with C-peptide across all logistic regression models. For each unit increase in D5D, individuals were approximately 0.73 times as likely across all models, to have serum C-peptide concentrations in the third tertile compared with the first tertile.

**Table 5:** The third tertile of serum C-peptide concentrations are significantly associated with PPL AA and D5D EAE^a^.

	Model 1 Age		Model 2 Age + BMI		Model 3 Age + WC	
Fatty Acid	OR (95% CI)	p-value	OR (95% CI)	p-value	OR (95% CI)	p-value
PA	1.01 (0.86, 1.19)	0.887	0.90 (0.75, 1.08)	0.246	0.89 (0.74, 1.08)	0.233
SA	1.19 (0.95, 1.48)	0.124	1.10 (0.87, 1.40)	0.422	1.04 (0.82, 1.33)	0.742
LGCA	0.41 (0.16, 1.04)	*0*.*060*	0.49 (0.17, 1.40)	0.183	0.47 (0.16, 1.35)	0.163
NA	0.39 (0.17, 0.90)	**0.027**	0.56 (0.21, 1.47)	0.238	0.71 (0.26, 1.94)	0.507
ALA ^b^	1.11 (0.79, 1.56)	0.566	1.33 (0.89, 1.99)	0.163	1.48 (0.95, 2.32)	*0*.*086*
EPA	0.95 (0.51, 1.76)	0.866	1.30 (0.64, 2.61)	0.466	1.85 (0.87, 3.96)	0.112
DHA	1.01 (0.73, 1.41)	0.937	1.17 (0.80, 1.70)	0.418	1.28 (0.87, 1.89)	0.211
Total ω-3	0.97 (0.78, 1.20)	0.773	1.14 (0.89, 1.46)	0.316	1.23 (0.95, 1.59)	0.117
LA	0.91 (0.81, 1.03)	0.132	1.01 (0.88, 1.17)	0.850	1.02 (0.89, 1.18)	0.736
DGLA	1.82 (1.06, 3.13)	**0.030**	1.14 (0.62, 2.09)	0.680	1.04 (0.55, 1.94)	0.912
AA	0.91 (0.77, 1.06)	0.209	0.82 (0.68, 0.99)	**0.044**	0.78 (0.64, 0.95)	**0.014**
DTA ^b^	1.04 (0.77, 1.41)	0.804	0.73 (0.50, 1.06)	0.101	0.67 (0.44, 1.01)	*0*.*057*
DPA ω-6 ^b^	1.54 (1.06, 2.23)	**0.024**	1.19 (0.78, 1.81)	0.432	1.15 (0.76, 1.76)	0.512
Total ω-6	0.89 (0.79, 1.01)	*0*.*065*	0.90 (0.79, 1.04)	0.153	0.88 (0.77, 1.02)	0.093
ω-6:ω-3	1.00 (0.87, 1.16)	0.966	0.95 (0.81, 1.12)	0.557	0.91 (0.77, 1.08)	0.285
D5D	0.69 (0.52, 0.93)	**0.015**	0.76 (0.56, 1.04)	*0*.*082*	0.75 (0.55, 1.02)	*0*.*068*
D6D ^b^	3.16 (1.29, 7.71)	**0.012**	1.28 (0.47, 3.47)	0.625	1.09 (0.39, 3.01)	0.872

^a^ Models defined as: C-peptide = fatty acid + independent variable(s) next to model number. Fatty acids expressed as percent of total phospholipids. P-values bolded if p ≤ 0.05 and italicized if 0.05 < p ≤0.09.

^b^ Odds ratios calculated on the basis that there is a unit change of 0.1 for the respective beta coefficient for each given parameter.

## Discussion

In this cross-sectional study, we investigated associations between C-peptide, leptin, and adiponectin, and PPL FAs or EAEs in a population of 126 males (> 96% Caucasian) ages 48–65. Summaries of our most significant results for leptin, adiponectin, and C-peptide across models are provided in Table 6. Leptin was positively associated with PPL SA and the ω-6/ω-3 ratio (Table 6). However, significant associations between PPL FAs and leptin faded across tertiles, after adjusting for BMI and WC (Table 6). Elevated concentrations of adiponectin were positively associated with PPL LA, and elevated concentrations of adiponectin were inversely associated with the D6D EAE. C-peptide was inversely associated with PPL DTA and the D5D EAE (Table 6). Together these data indicate specific PPL FAs and EAEs are associated with adiponectin and C-peptide concentrations even after adjusting for BMI and WC. These EAEs are generally reflective of FA metabolism, however, they may not completely represent enzyme kinetics in tissues. Therefore, reported altered EAEs could be related to other factors such as diet. We did not directly collect or assess dietary intake in this study, however, we used PPL FAs in our analysis which are correlated with dietary fat intake [26–28]. In addition, we recognize that the generalizability of these observations is limited and should be verified in larger populations.

**Table 6:** Summarized logistic regression results of adipokines and C-peptide associations with plasma phospholipid fatty acids and enzymes activity estimates.

PPL FA / EAE	Serum Parameter	Model 1 Age	Model 2 Age + BMI	Model 3 Age + WC
	**Leptin**			
SA	< 4.60 ng/mL	ref	ref	ref
	> 4.75 and ≤ 9.01 ng/mL	↑	↑	↑
	≥ 9.26 ng/mL	**↑**	**↑**	-
	**Leptin**			
ω-6/ω-3	< 4.60 ng/mL	ref	ref	ref
	> 4.75 and ≤ 9.01 ng/mL	↑	↑	-
	≥ 9.26 ng/mL	↑	-	-
	**Adiponectin**			
LA	≤ 3.62 μg/mL	ref	ref	ref
	> 3.64 and ≤ 5.37 μg/mL	-	-	-
	> 5.37 μg/mL	↑	↑	↑
	**Adiponectin**			
D6D	≤ 3.62 μg/mL	ref	ref	ref
	> 3.64 and ≤ 5.37 μg/mL	-	-	-
	> 5.37 μg/mL	↓	↓	↓
	**C-peptide**			
DTA	≤ 1.76 ng/mL	ref	ref	ref
	> 1.80 and ≤ 3.29 ng/mL	-	↓	↓
	≥ 3.32 ng/mL	-	-	↓
	**C-peptide**			
D5D	≤ 1.76 ng/mL	ref	ref	ref
	> 1.80 and ≤ 3.29 ng/mL	↓	↓	↓
	≥ 3.32 ng/mL	↓	↓	↓

Serum parameters are bolded, followed by concentrations for tertile 1, 2, and 3 for each respective adipokine or C-peptide. Ref indicates reference category used in the logistic regression model. ↑ represents increased odds and ↓ represents decreased odds, compared with the reference category, and are only provided if the p-value ≤0.09.

A–indicates no difference compared with the reference category

Currently there are no clinically defined biological cutoffs for circulating adiponectin or C-peptide concentrations. However, previous researchers have used tertiles to investigate associations with C-peptide concentrations [29] and adiponectin [30] in their study populations. We report PPL FAs and EAE differences were specific to adiponectin tertile. Inflammation suppresses adiponectin expression and secretion [31]. ω-6 PUFAs are considered “pro-inflammatory like”, especially AA derived eicosanoids. AA can be endogenously synthesized through elongation and desaturation of LA. Here we show PPL LA was positively associated with greater adiponectin concentrations (Table 3). Despite most ω-6 PUFAs having an inverse relationship with adiponectin when analyzed individually, total ω-6 PUFAs were positively associated with adiponectin (Table 3 and S1 Table). LA is elongated and desaturated to form DGLA, which is dependent on the enzyme D6D. Inflammation is positively associated with the D6D enzyme, and inhibiting D6D reduces inflammation [32]. The D6D EAE is reflective of D6D enzyme activity in several tissues, and the D6D EAE is calculated as the ratio of DGLA/LA (reviewed in detail [12]). We have previously shown D6D EAE is highly associated with both BMI and WC [24]. In this study, the third tertile of adiponectin was inversely associated with the D6D EAE after controlling for BMI and WC. This result was expected since individuals with adiponectin concentrations in the third tertile would likely have less inflammation compared with those with adiponectin concentrations in the first tertile. Increased D6D activity may promote inflammation in a way, which contributes to inhibiting adiponectin expression and/or decreasing adiponectin concentrations. Future studies should investigate the effects of altered cellular D6D enzyme activity on adiponectin expression and secretion.

Elevated concentrations of PPL AA and D5D EAEs were inversely associated with the second and third tertile of C-peptide after adjusting for BMI and WC (Tables 4 and 5). The D5D EAE is calculated as the ratio of AA/DGLA (reviewed in detail [12]). Our D5D EAE results indicate as C-peptide concentrations increase there is a greater amount of DGLA relative to AA. However, DGLA was only associated with C-peptide in the age-adjusted model (Table 5). DGLA is considered to be more “anti-inflammatory like”, compared with AA [33]. Decreased AA production would limit the substrate availability of AA for pro-inflammatory eicosanoid biosynthesis or elongation to form longer chain ω-6s (i.e. DTA). We report PPL DTA was inversely associated with C-peptide concentrations after adjusting for BMI and WC (Tables 4 and 5). From our point of view, the PPL DTA observation strengthens our speculation that FA metabolism (i.e. D5D) is altered in individuals with elevated C-peptide concentrations.

In this study we identify new relationships between C-peptide and D5D EAEs. What is unique about our current study is we associate C-peptide with D5D EAEs in a population of obese adults. Previous research has shown D5D EAEs are inversely associated with obesity, insulin resistance, T2D, and metabolic syndrome (reviewed in detail [12]). Our patient exclusion criteria excluded individuals with T2D. We initially speculated that the inverse relationship with C-peptide and D5D EAEs was likely due to underlying obesity-associated inflammation, since inflammation (i.e. TNF-α) alters insulin downstream signaling [16]. However, C-peptide concentrations were inversely associated with D5D EAEs after adjusting for age and TNF-α (data not shown). We have previously reported inflammatory factors such as TNF-α are highly correlated to BMI [24]. Therefore, TNF-α could not be analyzed in BMI and WC adjusted models due to multicollinearity with these anthropometric measurements. We found that TNF-α was not correlated with D5D EAEs (p-value = 0.9, data not shown), but TNF-α was positively correlated with C-peptide (p<0.001, data not shown). This led us to speculate C-peptide could be a potential regulator of D5D enzyme activity or expression.

Insulin functions in regulating D5D and D6D enzyme expression (reviewed in detail [34]). For instance, in insulin-dependent (i.e. type-1-diabetes (T1D)) animal models, D5D and D6D mRNA increase after insulin injections [35]. However, desaturase enzymes are not altered in T2D models, thus, the results are not consistent with T1D models. Montanaro et al reported there are no alterations to D5D and D6D mRNA or EAEs in insulin-independent (i.e. T2D) animals compared with control animals [36]. There is an overwhelming amount of human data associating increased D6D EAEs and decreased D5D EAES with obesity, insulin resistance, T2D, and metabolic syndrome (reviewed in detail [12]). One difference between T1D and T2D models is the endogenous production of pro-insulin and, thus, C-peptide. The lack of C-peptide in T1D models may account for increases in D5D mRNA and EAEs that were observed after insulin injections. Recent findings suggest C-peptide is able to activate signaling pathways associated with FA metabolism, independent of insulin [37]. Therefore, greater concentrations of C-peptide could be suppressing D5D enzyme activity or expression, which would explain why numerous researchers report D5D EAEs are inversely associated with obesity, insulin resistance, T2D, and metabolic syndrome (reviewed in detail [12]). Thus, it is possible C-peptide may function in regulating D5D enzyme activity. Future studies should investigate whether D5D inhibition may occur through a C-peptide mediated pathway.

## Supporting Information

S1 Table Plasma phospholipid fatty acids and enzyme activity estimates are associated with serum adipokines and C-peptide. (DOCX)

## Abbreviations

WAT: White adipose tissue
FA: Fatty acid
T2D: Type-2 diabetes
SatFAs: Saturated fatty acids
MUFAs: Monounsaturated fatty acids
PUFAs: Polyunsaturated fatty acids
ω-3: Omega-3
ω-6: Omega-6
PA: Palmitic acid
SA: Stearic acid
TNF-α: Tumor necrosis factor-α
EAE: Enzyme activity estimates
PL: Phospholipids
FAMEs: Fatty acid methyl esters
PPL: Plasma phospholipids
D5D: Delta-5-desaturase
D6D: Delta-6-desaturase
AA: Arachidonic acid
DGLA: Dihomo-γ-linolenic acid
LA: Linoleic acid
ALA: Alpha-linolenic acid
EPA: Eicosapentaenoic acid
DPA: Docosapentaenoic acid
DHA: Docosahexaenoic acid
DTA: Docosatetraenoic acid
OR: Odds ratio
BMI: Body mass index
WC: Waist circumference
T1D: Type-1 diabetes
