# Supplementary material for: Relationship between Body Mass Index, C-Peptide, and Delta-5-Desaturase Enzyme Activity Estimates in Adult Males
Source: PLoS One. 2016 Mar 29;11(3):e0149305. doi: 10.1371/journal.pone.0149305 (PMC4811535; doi:10.1371/journal.pone.0149305)
Supplement: S1 Table — (DOCX) [file pone.0149305.s001.docx]

**S1 Table: Plasma phospholipid fatty acids and enzyme activity estimates are associated with serum adipokines C-peptide ^a^**

| **Fatty Acid/ EAE** | **Leptin** | **Adiponectin** | **C-peptide** |
| --- | --- | --- | --- |
| PA | 0.026 | -0.221 | -0.068 |
|  | 0.7691 | **0.0128** | 0.4491 |
| SA | 0.313 | -0.080 | 0.236 |
|  | **0.0004** | 0.3745 | **0.0077** |
| LGCA | -0.120 | 0.022 | -0.175 |
|  | 0.1816 | 0.8057 | **0.0498** |
| NA | -0.354 | 0.130 | -0.251 |
|  | **<.0001** | 0.1456 | **0.0046** |
|  |  |  |  |
| ALA | -0.221 | 0.118 | 0.0165 |
|  | **0.0131** | 0.1894 | 0.8549 |
| DHA | -0.114 | 0.027 | 0.007 |
|  | 0.2039 | 0.7617 | 0.9423 |
| EPA | -0.279 | 0.106 | -0.111 |
|  | **0.0016** | 0.2371 | 0.2152 |
| DPAω-3 | -0.172 | **0.049** | -0.163 |
|  | *0.0541* | 0.5854 | *0.0688* |
| Total ω-3 | -0.203 | 0.074 | -0.351 |
|  | **0.0225** | 0.4133 | 0.6967 |
|  |  |  |  |
| LA | -0.185 | 0.284 | -0.182 |
|  | **0.0383** | **0.0012** | **0.0412** |
| DGLA | 0.432 | -0.181 | 0.263 |
|  | **<0.0001** | **0.0430** | **0.0029** |
| AA | 0.125 | -0.022 | -0.062 |
|  | 0.1644 | 0.8113 | 0.4927 |
| DTA | 0.267 | -0.192 | 0.050 |
|  | **0.0025** | **0.0314** | 0.5789 |
| DPAω-6 | 0.344 | -0.223 | 0.222 |
|  | **<0.0001** | **0.0122** | **0.0125** |
| Total ω-6 | 0.033 | 0.184 | -0.152 |
|  | 0.7145 | **0.0394** | *0.0895* |
|  |  |  |  |
| ω-6:ω-3 ratio | 0.193 | 0.027 | -0.013 |
|  | **0.0307** | 0.7667 | 0.8878 |
| D6D | 0.422 | -0.288 | 0.293 |
|  | **<.0001** | **0.0011** | **0.0009** |
| D5D | -0.275 | 0.143 | -0.263 |
|  | **0.0018** | 0.1104 | **0.0029** |

^a^ Spearman correlations between fatty acids, leptin, adiponectin, and C-peptide. White rows represent corresponding correlation coefficient, gray rows directly underneath represent corresponding p-value. p-values bolded if p ≤ 0.05 and italicized if 0.05 < p ≤0.09.
